# Engineering a Novel Bivalent Oral Vaccine against Enteric Fever

**DOI:** 10.3390/ijms22063287

**Published:** 2021-03-23

**Authors:** Annelise Soulier, Claudia Prevosto, Mary Chol, Livija Deban, Rocky M. Cranenburgh

**Affiliations:** Prokarium Ltd., London Bioscience Innovation Centre, 2 Royal College Street, London NW1 0NH, UK; annelise@deepscienceventures.com (A.S.); claudia.prevosto@prokarium.com (C.P.); mary.chol@prokarium.com (M.C.); rocky.cranenburgh@outlook.com (R.M.C.)

**Keywords:** bacteria, salmonella, Typhi, Paratyphi A, enteric fever, vaccine, synthetic biology

## Abstract

Enteric fever is a major global healthcare issue caused largely by *Salmonella enterica* serovars Typhi and Paratyphi A. The objective of this study was to develop a novel, bivalent oral vaccine capable of protecting against both serovars. Our approach centred on genetically engineering the attenuated *S.* Typhi ZH9 strain, which has an excellent safety record in clinical trials, to introduce two *S.* Paratyphi A immunogenic elements: flagellin H:a and lipopolysaccharide (LPS) O:2. We first replaced the native *S.* Typhi *fliC* gene encoding flagellin with the highly homologous *fliC* gene from *S.* Paratyphi A using Xer-cise technology. Next, we replaced the *S.* Typhi *rfbE* gene encoding tyvelose epimerase with a spacer sequence to enable the sustained expression of O:2 LPS and prevent its conversion to O:9 through tyvelose epimerase activity. The resulting new strain, ZH9PA, incorporated these two genetic changes and exhibited comparable growth kinetics to the parental ZH9 strain. A formulation containing both ZH9 and ZH9PA strains together constitutes a new bivalent vaccine candidate that targets both *S.* Typhi and *S.* Paratyphi A antigens to address a major global healthcare gap for enteric fever prophylaxis. This vaccine is now being tested in a Phase I clinical trial (NCT04349553).

## 1. Introduction

Enteric fever is an infectious disease that causes significant mortality and morbidity in low- and middle-income countries (LMICs), such as those in South Asia and sub-Saharan Africa [1,2,3]. Enteric fever is predominantly caused by two serotypes of *Salmonella enterica*: *S.* Typhi, which is responsible for around 10.9 million cases per year, and *S.* Paratyphi A*,* which is responsible for around 3.4 million cases per year, based on 2017 data [2].

The widespread use of antibiotics in LMICs has led to the emergence of extensively drug-resistant (XDR) *Salmonella* strains that are no longer susceptible to multiple lines of antibiotics. For example, in a recent outbreak across three sites in Pakistan, over 90% of both *S.* Typhi and *S.* Paratyphi A isolates were resistant to fluoroquinolone [4]. Consequently, the World Health Organization (WHO) has listed fluoroquinolone-resistant *Salmonellae* as high priority pathogens for the research and development of new antibiotics [5].

Several vaccines are currently licensed for *S.* Typhi, including a live attenuated oral vaccine (Vivotif™) and parenteral injectable vaccines [6]. However, these do not protect against the *S.* Paratyphi A strain [7], the prevalence of which is increasing [8]. For example, two hospital-based studies in Nigeria found that a considerable proportion of enteric fever cases (ranging from 17 to 34%) were attributable to *S*. Paratyphi A [9,10]. Furthermore, several sources agree that enteric fever—in particular, that caused by *S.* Paratyphi A—is almost certainly under-reported in LMICs [11,12]. Therefore, the development of a bivalent vaccine that protects against both *S.* Typhi and *S.* Paratyphi A would address a serious global healthcare need.

Using a live attenuated orally administered vaccine has several potential advantages over injectable subunit Vi vaccines for protecting against enteric fever. These include a longer duration of protection, the establishment of immunological memory and a reactive immune profile that more closely follows that of natural infection [13]. Several groups have constructed live attenuated *S*. Paratyphi A vaccines. For example, an oral dose of *S*. Paratyphi A (Δ*phoPQ*) was well tolerated in rabbits [14], and an oral dose of *S*. Paratyphi A CVD 1902 (Δ*guaBA*, Δ*clpX*) was immunogenic in healthy volunteers [15]. However, these attenuated *S*. Paratyphi A strains have very limited safety data available in comparison with attenuated *S*. Typhi strains and do not offer simultaneous protection against both causative agents of enteric fever.

We have taken the approach of engineering a pre-existing oral attenuated Salmonella vaccine technology to enable its rapid adaptation against new disease targets. In clinical formulations, this vaccine is simple to manufacture and is extremely cost-effective. The parental vaccine strain, *S.* Typhi ZH9, has previously been shown to be safe in multiple clinical trials across a wide range of participant populations, including children living in an endemic LMIC [16,17,18,19,20,21]. In this study, we describe the process of engineering a new *S.* Typhi strain expressing two *S.* Paratyphi A antigens (LPS O:2 and H:a flagellin) that can be formulated in equivalent amounts with the *S.* Typhi ZH9 parental strain to create a bivalent vaccine designed to provide protection against both *S.* Typhi and *S.* Paratyphi A. We present data describing the genetic engineering, *in vitro* characterisation and *in vivo* immunogenicity of the basic formulation of a new bivalent enteric fever vaccine named Entervax™, which is now in clinical testing (NCT04349553) [22].

## 2. Results

### 2.1. Converting Flagellin from H:d to H:a

**Figure 1:**
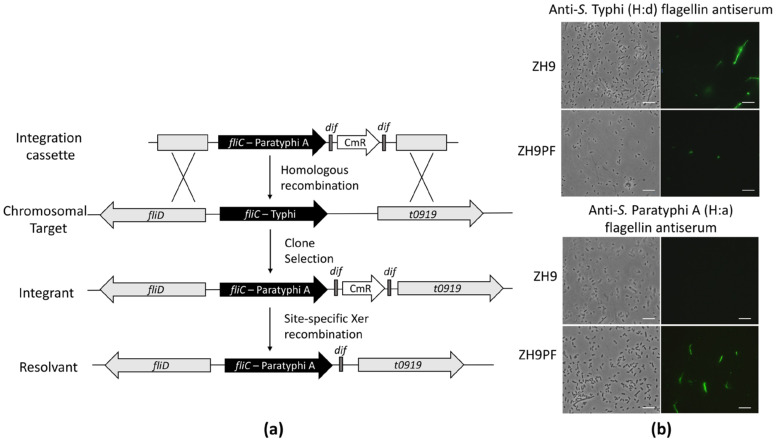
Replacing the *S.* Typhi (H:d) flagellin with *S.* Paratyphi A (H:a) flagellin. (**a**) The genetic engineering process to generate *S.* Typhi ZH9 expressing *S.* Paratyphi A flagellin (ZH9PF). Adapted with permission from Bloor and Cranenburgh, 2006 [23]. (**b**) Fluorescence microscopy with *S.* Typhi ZH9 and the derivative strain, ZH9PF, probed with H:d antiserum (anti-*S.* Typhi) or H:a antiserum (anti-*S.* Paratyphi A) plus Dylight 488 secondary antibodies; the left column images are phase contrast images, and the right column images are immuno-fluorescence images. Images were taken at 100× magnification. Scale bars represent 10 µm. Representative images were based on three independent experimental repeats.

The construction of the parental *S.* Typhi ZH9 has previously been described, and it represents an attenuated strain with Δ*aroC* and Δ*ssaV* mutations [17]. To begin constructing a hybrid *S.* Typhi strain expressing two immunogenic elements from *S.* Paratyphi A, we first converted the parental ZH9 H:d serotype to a H:a serotype by replacing the native *fliC* gene encoding flagellin with the *S.* Paratyphi A *fliC* gene (Figure 1a; see the methods section for more information).

The successful conversion of the H:d flagellin serotype to H:a in ZH9PF was tested by immunostaining. Bacteria were incubated with H:d antiserum or H:a antiserum plus fluorescent secondary antibodies, followed by visualisation with fluorescence microscopy. The parental ZH9 strain was not reactive against anti-*S.* Typhi (H:a) flagellin antiserum, while the modified ZH9PF strain showed positive staining (Figure 1b). Conversely, the parental ZH9 strain was reactive against anti-*S.* Typhi (H:d) flagellin antiserum, while the modified ZH9PF strain was minimally reactive (Figure 1b). The minimal fluorescence observed after staining ZH9PF with H:d flagellin antiserum appeared to be restricted to the membrane, suggesting that this was due to the polyclonal H:d serum recognising surface proteins in addition to its designated target, flagellin. Overall, it was clear that swapping the *fliC* gene successfully converted the flagellin expressed by ZH9 from an H:d to H:a serotype.

### 2.2. Modifying LPS from O:9 to O:2

**Figure 2:**
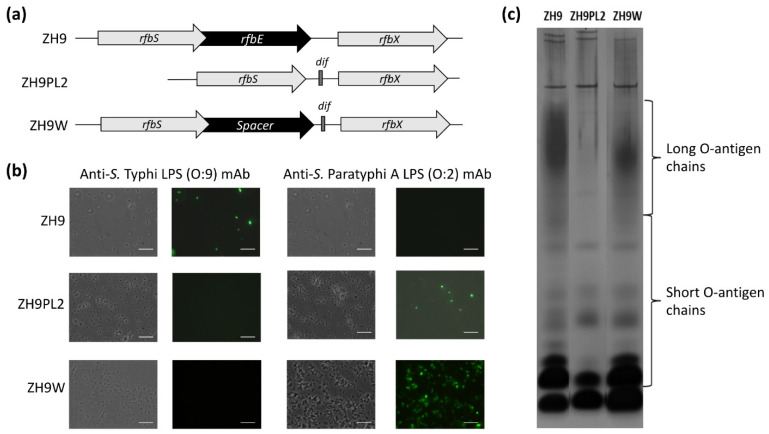
Modifying LPS (O:9) to LPS (O:2). (**a**) Part of the wild-type O-antigen locus from *S.* Typhi ZH9 was modified using two test approaches: by deleting the majority of the *rfbE* cistron to generate *S.* Typhi ZH9PL2 or by replacing the *rfbE* cistron with a spacer DNA sequence to maintain the original reading frame to generate *S.* Typhi ZH9W. (**b**) Fluorescence microscopy images showing the parental *S.* Typhi ZH9 and derivative strains, ZH9PL2 and ZH9W, probed with anti-*S.* Typhi LPS (O:9) or anti-*S.* Paratyphi A LPS (O:2) monoclonal antibodies followed by Dylight 488 secondary antibodies; the left column images are phase contrast images and the right column images are immuno-fluorescence micrographs. Images were taken at 100× magnification. Scale bars represent 10 µm. Representative images based on three independent experimental repeats. (**c**) Silver-stained polyacrylamide gel of LPS extracts from the parental *S.* Typhi ZH9 and derivative strains, ZH9PL2 and ZH9W, indicating the short and long O-antigen chains. LPS = lipopolysaccharide; mAb = monoclonal antibody.

Using the parental ZH9 strain, we next tested two different approaches to convert the native *S.* Typhi LPS O:9 antigen to the *S.* Paratyphi A O:2 antigen. The CDP-d-tyvelose 2-epimerase (Tyv) of *S.* Typhi, encoded by the *rfbE* gene, catalyses the isomeration of 3,6-dideoxyhexose sugars CDP-paratose to CDP-tyvelose, which is incorporated in the O-antigen, thus conferring serogroup specificity O:9. In *S.* Paratyphi A, the *rfbE* gene is mutated, so paratose is incorporated in the O-antigen instead, thus conferring serogroup specificity O:2 [24]. In our first approach, the majority of the *rfbE* gene was simply deleted; in our second approach, the *rfbE* gene was fully replaced with the spacer gene, *wbdR*, which encodes a putative *N*-acetyltransferase gene that is not functional in *Salmonella* (Figure 2a). Both approaches followed a similar process for generating a chromosomal integration cassette, as described above for switching *fliC* genes. 

In the first approach to delete the majority of the *rfbE* gene, the *dif*-flanked antibiotic resistance gene, *cat,* was amplified with primers rfbEdelF and rfbEdelR, which comprised homologous sequences to *rfbS* and *rfbX* genes, respectively, to generate ZH9PL2. In the second approach to fully replace *rfbE* with the *wbdR* spacer cistron, we synthesised the full *wdbR* spacer cistron, flanked on one side by approximately 700 bp of DNA homologous to the *S.* Typhi *rfbE* upstream gene, *rfbS*, and on the other side, by approximately 700 bp of DNA homologous to the *S.* Typhi downstream gene, *rfbX,* to create a deletion cassette and generate ZH9W (Figure 2a). 

The conversion of the O:9 to the O:2 serotype of LPS in both ZH9PL2 and ZH9W was tested by direct staining of bacteria with anti-*S.* Typhi LPS (O:9) or anti-*S.* Paratyphi A LPS (O:2) monoclonal antibodies (mAbs), followed by visualisation using fluorescence microscopy. Both the ZH9PL2 and ZH9W strains were reactive against the O:2 mAb but not the O:9 mAb, suggesting that both approaches had successfully converted LPS from O:9 to O:2 (Figure 2b). The parental ZH9 strain was reactive against the O:9 mAb but not the O:2 mAb, as expected (Figure 2b). However, SDS-PAGE silver-stain analysis of ZH9PL2 and ZH9W LPS extracts highlighted that only when the *rfbE* gene had been replaced by the spacer gene, *wbdR,* was LPS expressed with long O-antigen chains (Figure 2c; Figure S1). Thus, we concluded that the second approach—to replace *rfbE* with a *wbdR* spacer gene—generated a strain that was functionally the closest match to the parental strain.

### 2.3. Constructing the Final New Strain, ZH9PA

To generate the final ZH9PA strain incorporating both the H:d-to-H:a flagellin and O:9-to-O:2 LPS modifications, the ZH9PF strain (containing the *fliC* gene replacement) was further modified to replace the *rfbE* gene with the spacer gene, *wbdR*. These modifications of the LPS and flagellin loci were verified by sequencing (data not shown), and the expression of LPS and flagellin was analysed using immunostaining followed by fluorescence microscopy, western blot and silver staining techniques. 

**Figure 3:**
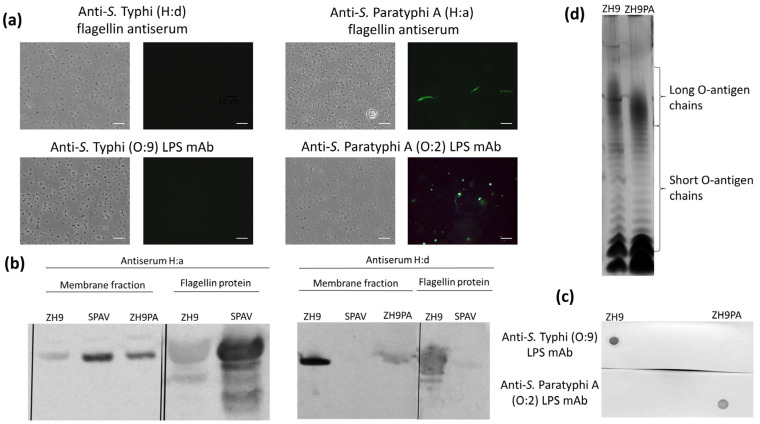
Converting flagellin and LPS in the final new strain, ZH9PA. (**a**) Fluorescence microscopy images showing the *S.* Typhi ZH9 derivative strain, ZH9PA, probed with anti-*S*. Typhi (H:d) or anti-*S*. Paratyphi A (H:a) flagellin antiserum and anti-*S.* Typhi (O:9) or anti-*S.* Paratyphi A (O:2) LPS mAbs; the left images are phase contrast images and right images are immuno-fluorescence micrographs. Images were taken at 100× magnification. Scale bars represent 10µm. Representative images based on three independent experimental repeats. (**b**) Western blots of membrane fractions probed with anti-*S.* Typhi (H:d) or anti-*S.* Paratyphi A (H:a) flagellin antisera using ZH9 or SPAV as positive controls, respectively. Purified flagellin proteins were also included as a positive control. (**c**) Dot blot probed with anti-*S.* Typhi and anti-*S.* Paratyphi A LPS mAbs. (**d**) Silver-stained polyacrylamide gel of LPS preparations from *S.* Typhi ZH9 and derivative strains, ZH9PA, indicating the short and long O-antigen chains. LPS = lipopolysaccharide; mAb = monoclonal antibody; SPAV = attenuated *S.* Paratyphi A.

Based on immunostaining, ZH9PA was reactive against both *S.* Paratyphi A LPS (O:2) and flagellin (H:a) targets but not *S.* Typhi LPS (O:9) or flagellin (H:d) (Figure 3a). Similarly, by western blot, the bacterial membrane fractions from ZH9PA were positive for *S.* Paratyphi A flagellin H:a but only weakly positive for *S.* Typhi flagellin H:d (Figure 3b; Figure S2). Heat-inactivated bacterial preparations from ZH9PA were also positive for LPS O:2 (but not LPS O:9) by dot blot (Figure 3c; Figure S3). Finally, silver-stained SDS-PAGE analysis confirmed long O-chain expression in ZH9PA, as well as in the ZH9 parent vaccine strain (Figure 3d).

### 2.4. Evaluating the Growth of ZH9PA

We tested whether the introduction of the *S.* Paratyphi A flagellin (H:a) and the replacement of *S.* Typhi *rfbE* with a spacer gene had an impact on ZH9PA bacterial growth in culture compared to the parental ZH9 strain. For both strains, an OD_600 nm_ = 0.1 was seeded into liquid culture and allowed to grow for 24 h. At regular intervals, samples were taken, and counts were made using both optical density (OD_600 nm_) and colony-forming unit (CFU/mL) measurements.

**Figure 4:**
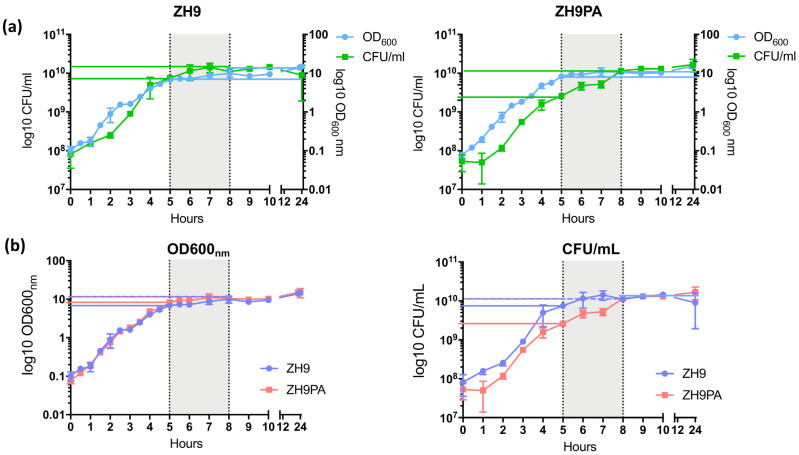
Comparison of growth profiles. Bacteria were seeded into LB broth cultures at OD_600 nm_ = 0.1 and grown for 24 h. At regular intervals, samples were taken and analysed by spectrophotometry or by titration on agar plates. Optical density (a measure of growth density) and bacterial titre were plotted for ZH9 (the parental strain) and ZH9PA (the modified strain). The late exponential growth phase (5 to 8 h) is shown in grey. (**a**) OD_600 nm_ and CFU/mL measurements compared within each individual strain. (**b**) OD_600 nm_ or CFU/mL measurements compared between both strains. Statistical comparisons were made using a two-way ANOVA, based on triplicate cultures in a single experiment. CFU = colony-forming units; ml = millilitres; nm = nanometres.

We first compared OD_600 nm_ vs. CFU/mL within each individual bacterial strain. The patterns of growth observed over time using both methods suggested that ZH9PA had a slight growth delay together with a longer lag phase compared to the parental ZH9 strain; although by 8 h (the end of the late exponential growth phase, defined as between 5 to 8 h of culture), both strains achieved similar colony counts (1.1 × 10^10^ vs. 1.15 × 10^10^ CFU/mL for ZH9 vs. ZH9PA, respectively) (Figure 4a).

Next, we compared OD_600 nm_ or CFU/mL between the two strains (ZH9 vs. ZH9PA). In the late exponential growth phase, the optical density at 600 nm was similar for both strains. This ranged from 7 to 10 for the parental ZH9 strain and from 8 to 11 for the modified ZH9PA strain (Figure 4b). Colony counts within the late exponential growth phase were generally higher for the parental ZH9 strain, ranging from 7.5 × 10^9^ to 1.43 × 10^10^ for ZH9 and from 2.6 × 10^9^ to 1.15 × 10^10^ for ZH9PA strain (Figure 4b). No statistically significant differences were observed between the two strains based on either approximate bacterial cell counts (optical density measurements) or absolute bacterial cell counts (CFU/mL) at any timepoint (Figure 4b). Overall, we concluded that the two modifications present in strain ZH9PA had a negligible impact on strain fitness compared to the parental ZH9 strain.

### 2.5. Evaluating the Immunogenicity of the Basic Bivalent Enteric Fever Vaccine

We generated a basic vaccine formulation that mixed equivalent amounts of the ZH9 parental strain and the new ZH9PA strain to create a bivalent vaccine designed to generate immune responses against both *S.* Typhi and *S.* Paratyphi A bacteria. In order to assess antibody responses elicited against LPS antigens, and to ensure that the combination of two strains did not impair the response to individual LPS antigens, several groups of naive Balb/c mice were vaccinated subcutaneously with 1 × 10^8^ CFU of ZH9, 1 × 10^8^ CFU of ZH9PA or a basic Entervax™ formulation (a 1:1 combination of 0.5 × 10^8^ CFU of ZH9 and 0.5 × 10^8^ CFU of ZH9PA). Serum samples were obtained before vaccination (d0) and at 35 or 42 days following vaccination and analysed for anti-LPS O:9 or O:2 IgG antibody responses using enzyme-linked immunosorbent assays (ELISA).

**Figure 5:**
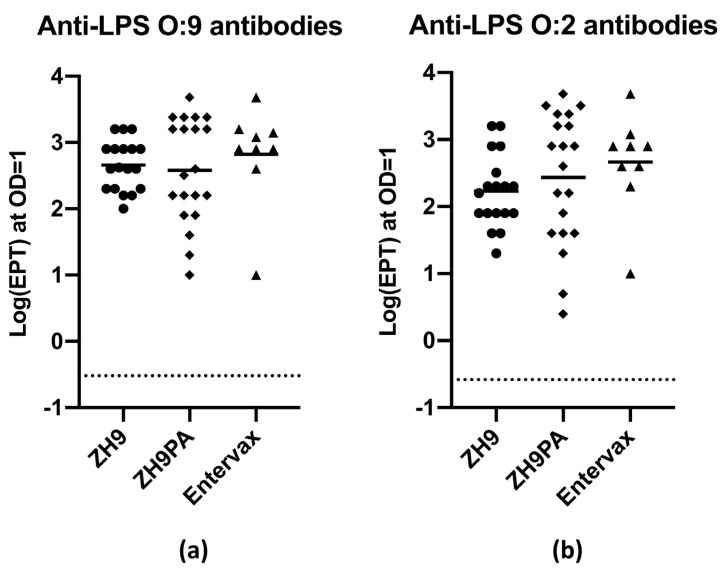
Anti-LPS IgG antibody responses following *in vivo* vaccination. (**a**) Specific IgG antibody responses against *S.* Typhi LPS (O:9). (**b**) Specific IgG antibody responses against *S.* Paratyphi A LPS (O:2). Antibody responses were evaluated by ELISA in Balb/c mouse serum at 35 or 42 days following subcutaneous vaccination with 10^8^ CFU ZH9 (•), 10^8^ CFU ZH9PA (♦) or a 1:1 mix of 0.5 × 10^8^ CFU of ZH9 and 0.5 × 10^8^ CFU of ZH9PA (Entervax™ basic formulation (▲)). Pre-vaccination (d0) samples were pooled across individual mice to generate the negative assay control (dotted line). Each data point represents an individual mouse, and data were pooled across three independent experiments. Mean values are represented by the horizontal bar. Statistical comparisons were made using a one-way ANOVA. ELISA = enzyme-linked immunosorbent assay; EPT = end point titre; IgG = immunoglobulin G; LPS = lipopolysaccharide; OD = optical density.

By ELISA, it was clear that in mice receiving the dual vaccine combination, both the parental ZH9 strain and the new ZH9PA strain could generate equivalent anti-LPS IgG responses against LPS O:9 and O:2, respectively (Figure 5). Importantly, this provided evidence that both strains could be administered together without one strain outcompeting the other in terms of antigenic dominance. Mice vaccinated with each strain individually were also capable of mounting robust IgG responses against LPS targets (Figure 5). Due to the high level of sequence homology between O:9 and O:2 LPS proteins, it was not possible to fully discriminate between the specific anti-O:9 or anti-O:2 antibody responses using currently available antigens and reagents. However, overall, we concluded that immunocompetent mice could mount equivalent immunogenic IgG antibody responses against both ZH9 and ZH9PA, making the combination a promising bivalent vaccine candidate for the prevention of enteric fever.

## 3. Discussion

We have described a two-step process to genetically engineer a novel strain of *Salmonella* that has the genotype of attenuated *S.* Typhi ZH9 but has been modified to express the flagellin and LPS of *S.* Paratyphi A. This novel strain, ZH9PA, showed similar growth kinetics to the parental ZH9 strain, with a negligible impact on strain fitness. When ZH9PA was administered in equivalent proportions with ZH9 to create the basic formulation for a new vaccine, Entervax™, immunogenic IgG antibody responses were observed against both ZH9 (LPS O:9) and ZH9PA (LPS O:2) following subcutaneous vaccination of immunocompetent mice.

Several groups have attempted to develop vaccines that can protect against either *S.* Typhi or *S.* Paratyphi A, but few have focussed on developing a vaccine that protects against both. Those that have pursued a bivalent approach have typically introduced changes to both *S.* Typhi and *S.* Paratyphi wild-type strains, requiring safety and environmental validation of two independent strains [25]. Through targeted engineering of the ZH9 strain as the vaccine chassis, we have leveraged an existing profile of robust clinical safety with validated stable attenuating mutations (Δ*aroC*, Δ*ssaV*) to build a bivalent vaccine formulation capable of eliciting immune responses against both *S.* Typhi and *S.* Paratyphi A.

In our study, we used Xer-cise technology to successfully replace the *fliC g*ene encoding *S.* Typhi ZH9 flagellin with the *fliC* gene encoding *S.* Paratyphi A flagellin, and to remove the antibiotic resistance gene used to select the correct mutant from the bacterial chromosome. We used the same recombination technology to replace the *rfbE* gene encoding CDP-d-tyvelose 2-epimerase with the spacer gene, *wbdR*. Our results showed that simple deletion of *rfbE* was not sufficient to maintain the long-O antigens that were a critical feature of the parental strain. This may have been due to disruption of operon transcription, as downstream of *rfbE*, the gene *rfbX* encodes a Wzx translocase involved in the translocation of bacterial O-antigen repeat units across the cytoplasmic membrane [26,27]. These findings are in line with those described by Hong et al. 2012, where Wzx translocation was reported to be *Salmonella* serotype-specific for the repeat-unit structure, and variants with sugar differences were translocated with lower efficiency and minimal production of long-chain O antigens [28]. Therefore, we found it necessary to replace the deleted *rfbE* gene with a spacer gene to enable native generation of long-O antigens and maintain translocase activity.

The two *S.* Paratyphi A antigens that we introduced into *S.* Typhi ZH9 were carefully chosen as robust vaccine components: both the O-antigen from LPS and the H-antigen from flagellin are highly immunogenic and have been used as the basis for serotyping *Salmonella* serovars together with the Vi antigen as part of the Kauffmann–White scheme for many years [29]. Both are potent pathogen-associated molecular patterns (PAMPs) that stimulate innate immune responses [30]. The glycolipid LPS forms part of the outer leaflet of the exterior *Salmonella* bacterial membrane, and the O-antigen polysaccharide represents one of the two carbohydrate regions within the LPS structure [29,31,32]. Antibodies generated against O-antigens are highly protective against lethal *Salmonella* infections in mice [33,34,35]. Similarly, flagellin, a subunit of the bacterial flagellum that enables motility, chemotaxis and invasion, is a potent immune activator [30]. Mice treated with vaccines targeting flagellin proteins are also significantly protected against lethal *Salmonella* challenges [36,37,38].

There were some technical limitations that we noted in our study. During the initial testing and development of our ELISA assay designed to detect anti-LPS IgG antibodies, it was not possible to fully discriminate between specific anti-*S.* Typhi O:9 or anti-*S.* Paratyphi A O:2 antibody responses using currently available antigens and reagents. The LPS moieties of both *S.* Typhi (O:9) and *S.* Paratyphi A (O:2) share substantial homology, with only a single major moiety differing between LPS O:9 and LPS O:2 (tyvelose and paratose, respectively) [39]. Notwithstanding this lack of absolute specificity, it was clear from our results that both the individual ZH9 and ZH9PA strains as well as the combination of the two strains (ZH9+ZH9PA) generated anti-LPS O:9 and O:2 IgG responses. Similarly, we noted some cross-reactivity in our immunostaining assays designed to detect flagellin antigens. Bacteria that had been modified to express H:a flagellin (but retain their native LPS; the ZH9PF strain) showed a low level of positive membrane staining when probed with the anti-flagellin H:d antiserum. This suggested that the polyclonal antiserum was recognising surface proteins in addition to the key target, flagellin. This interpretation is supported by the loss of this positive staining when the final ZH9PA strain (where the native versions of both flagellin and LPS were absent) was probed with H:d antiserum. Nevertheless, it remained clear that swapping the *fliC* gene successfully converted the flagellin expressed by ZH9 from an H:d to H:a serotype. Since *S.* Typhi is restricted to human hosts only, we used subcutaneous immunisation as a proxy for validating systemic immune responses to the Entervax™ formulation in a mouse model; however, the oral route of administration will be pursued in clinical trials based on strong positive safety and immunogenicity data from the clinical testing of ZH9. Similarly, since both of the *Salmonella* strains targeted by our vaccine represent biosafety category 3 pathogens [40], we were unable to validate vaccine (antibody) functionality by serum bactericidal assay or opsonophagocytic killing assay; this will be tested in the clinical trial setting using patient material. Finally, it was also not possible to test vaccine protection in an *in vivo* challenge model, since *S.* Typhi is a human-specific pathogen, and no robust mouse models exist.

Overall, this study describes a novel bivalent vaccine capable of generating immunogenic antibody responses against both *S.* Typhi and *S.* Paratyphi A using a bacterial strain with an established safety record. Our *in vitro* data confirm the successful genetic engineering of this vaccine, and our *in vivo* data highlight the immunogenicity of this bivalent vaccine based on IgG antibody responses. Based on this evidence, we have now received regulatory acceptance to initiate a Phase I clinical trial to confirm the safety, tolerability and immunogenicity of the Entervax™ vaccine delivered as an oral formulation in healthy human volunteers (NCT04349553) [22]. This vaccine is positioned to address a major unmet need in global healthcare by providing a new technology that is capable of targeting both *S.* Typhi and the increasingly prevalent *S.* Paratyphi A for the prevention of enteric fever.

## 4. Materials and Methods

### 4.1. Bacterial Strains and Media

The target strain for chromosomal modifications was Prokarium’s proprietary *S.* Typhi strain ZH9 (Δ*aroC*, Δ*ssaV*) derived from *S*. Typhi Ty2 [17]. This strain was cultured in LB broth containing 2 mg/L 4-aminobenzoic acid, 2 mg/L 2,3-dihydroxybenzoic acid, 8 mg/L L-phenylalanine, 8 mg/L L-tryptophan and 8 mg/L L-tyrosine, and on 1.5% agar plates. SPAV was an attenuated *S*. Paratyphi A strain (ΔssaV) derived from *S.* enterica serotype Paratyphi A (NCTC 9322; ECACC, Porton Down, UK). Top10 *Escherichia coli* bacteria (Life Technologies, Paisley, UK) were used for routine cloning. LB and LB–aro mix media were supplemented with 20 µg/mL chloramphenicol to select transformants and chromosomal integrants. All bacterial cultures were incubated at 37 °C, with shaking at 200 revolutions per minute (RPM) for liquid cultures. The full list of strains and plasmids used in this study are described in Table 1.

### 4.2. Generation of Dif-Flanked Antibiotic Resistance Gene Insertion Cassette

The *de novo*-synthesised chloramphenicol acetyltransferase (*cat*) gene from pBRT1Nc was amplified by polymerase chain reaction (PCR) using Q5 polymerase at 1 unit/µL (NEB, Hitchin, UK) and 5NotIdifcat and 3NotIdifcat primers (diluted 1:10 in sterile water from a stock solution of 100 pmol/µL). The DifCAT cassette that was generated was cut with a NotI restriction enzyme (NEB, Hitchin, UK) and ligated to generate Xer-cise plasmids pUCpW_difCAT and pUCpF_difCAT using Quick-Stick Ligase (Bioline Reagents Limited, London, UK) and Top10 *E. coli* competent cells (see Results) [41]. These were also amplified with primers rfbEdelF and rfbEdelR (designed with homologous 5′ sequences to *rfbS* and *rfbX* genes, respectively) and Q5 polymerase (NEB, Hitchin, UK) to create a *rfbE* chromosomal deletion cassette.

### 4.3. Chromosomal Integration Procedure

Chromosomal replacement and gene deletion were carried out as previously described [23]. Briefly, *S.* Typhi ZH9 was first transformed with a pLGBK plasmid coding for λ Red gene functions for integration of linear DNA. Integration cassettes were manufactured by Oxford Genetics (Oxford, UK), linearised with SalI and SacI restriction enzymes, purified and concentrated using gel purification (Zymoclean, Irvine, CA, USA) to add a minimum of 300 ng DNA to 50 µL electrocompetent ZH9(pLGBK) or ZH9PF (pLGBK). After electroporation, cells were resuspended in 1 mL LB–aro mix containing 0.02% arabinose and incubated for 18 h at 37 °C and 200 RPM for recovery and integration. Cells were plated on LB–aro mix containing 20 μg/mL chloramphenicol. Colonies were screened by polymerase chain reaction (PCR) with primers designed on the genomic DNA outside the homologous sequences of the integration cassette using My Taq HS Red mix (Bioline Reagents Limited, London, UK). Positive clones were cultured overnight in 5 mL LB at 37 °C and 200 RPM to delete the *cat* gene using Xer-cise technology [41]. Glycerol stocks were made and stored at −80 °C, and modified chromosomal sequences were amplified with Q5 polymerase and checked by sequencing using the diagnostic primers listed in Table 2.

### 4.4. Conversion of Flagellin

The *fliC* replacement cassette was synthesised to comprise *S.* Paratyphi A *fliC*, flanked on one side by approximately 700 bp of DNA homologous to the *S.* Typhi *fliC* upstream gene, *fliD*, and on the other side by approximately 700 bp of DNA homologous to the *S.* Typhi *fliC* downstream *T0919* pseudogene DNA sequence. A NotI restriction site at the 3′ end of the *S.* Paratyphi A *fliC* gene was incorporated to enable insertion of the *dif*-flanked *cat* antibiotic resistance marker gene amplified with primers designed with a corresponding NotI restriction site. TOP10 *E. coli* bacteria were used for transformation to generate a pUCpF_difCAT plasmid. Chromosomal replacement of the *S.* Typhi *fliC* gene with *S.* Paratyphi A *fliC* was carried out as previously described [23]. Briefly, *S.* Typhi ZH9 was first transformed with a pLGBK plasmid coding for λ Red gene functions for integration of linear DNA. Electrocompetent ZH9(pLGBK) was transformed, and the replacement cassette was excised from pUCpF-difCAT using SalI and SacI restriction digestion. Transformed colonies were selected on LB–aro mix agar plates supplemented with 20 μg/mL chloramphenicol. Single colonies were isolated and cultured overnight in LB–aro mix broth in the absence of antibiotics. Xer recombination resulted in the deletion of the *cat* gene to generate chloramphenicol-sensitive colonies of ZH9PF.

### 4.5. Immunostaining

For immunofluorescence microscopy, a volume of bacterial culture equivalent to an optical density (OD) at A_600 nm_ = 1 was collected, centrifuged at 6000× *g* for 5 min, and washed in phosphate-buffered saline (PBS). Pellets were resuspended in 10 µL of PBS with 1 µL of primary antibody and incubated for 10 min at room temperature. Flagellin analysis was carried out by staining bacteria with H:d antiserum (SSI Diagnostica) or H:a antiserum (SSI Diagnostica, Oxford Biosystems, Oxford, UK). LPS analysis was carried out by staining bacteria with anti-*S.* Typhi LPS (clone B348M; GeneTex, Insight Biotechnology, Wembley, UK) or anti-*S.* Paratyphi A LPS (clone 10B10G; Bio-Rad Laboratories, Hemel Hempstead, UK) monoclonal antibodies. Stained bacterial cells were washed in PBS and pellets were resuspended in 10 µL of PBS with 1 µL of goat anti-mouse secondary antibody (for LPS; Sigma-Aldrich Merck Life Science UK Limited, Gillingham, UK) or goat anti-rabbit secondary antibody (for flagellin; Sigma-Aldrich) conjugated to the Dylight 488 fluorochrome (Bio-Rad Laboratories, Hemel Hempstead, UK) for 10 min at room temperature. Bacterial cells were subsequently washed in PBS and a small volume was applied onto microscope slides to be visualised using a fluorescent microscope (Zeiss Axiophot, Carl Zeiss AG, Oberkochen, Germany) with an attached Zeiss Axiocam camera.

### 4.6. Western and Dot Blot

To generate protein samples for western blot, bacterial cells were harvested by centrifugation at 10,000× *g* and 4 °C for 10 min, washed in 10 mM Tris Buffer pH 7.4, then sonicated for 4 min with 30 s bursts at 60% amplitude (Fisherbrand™ 120 Sonic Dismembrator 50/60 Hz (Fisher Scientific UK Ltd., Loughborough, UK)). An initial centrifugation was performed at 6000× *g* and 4 °C for 10 min to remove unbroken cells; and a second centrifugation was performed at 30,000× *g* and 4 °C for 45 min to separate the cell membranes and cytoplasmic fractions. Pelleted envelopes were resuspended in 10 mM Tris Buffer pH 7.4 plus 2% (*v*/*v*) Triton X-100 (VWR, Lutterworth, UK) and incubated at 25 °C for 15 min to allow solubilisation of the inner membrane. Extracted envelopes were collected by centrifugation at 30,000× *g* and 4 °C for 45 min. Pellets were washed and resuspend in 10 mM Tris Buffer pH 7.4. Cytoplasmic and membrane fractions were stored at −80 °C prior to western blot analysis. Flagellin H:d and H:a proteins were purified from ZH9 and SPAV strains, respectively (The Native Antigen Company, Kidlington, UK) and used as positive controls; flagellin proteins were stored at −80 °C. Protein separation by molecular mass was performed via sodium dodecyl sulphate–polyacrylamide gel electrophoresis (SDS-PAGE) using NuPAGE 4–12% Bis-Tris Protein Gels and MES running buffer (Life Technologies, Paisley, UK) following the manufacturer’s instructions. The SeeBlue™ Plus2 pre-stained protein standard (Life Technologies, Paisley, UK) was used as a molecular marker. Protein gels were transferred onto membranes using a semi-dry trans-blot turbo transfer system (Bio-Rad Laboratories, Hemel Hempstead, UK), when required.

To generate protein samples for dot blot, bacteria were grown at 37 °C for 18 h. 0.5 mL of all tested cultures were heat-inactivated at 95 °C for 10 min. 5 μL of heat-inactivated cultures were dotted onto nitrocellulose membranes.

Membranes were incubated with 5% skim milk diluted in PBS for 1 h at room temperature to minimise non-specific antibody binding prior to adding primary antibodies following the same staining protocol described in the ‘Immunostaining’ section. After three washes with 0.05% Tween-20 (VWR, Lutterworth, UK) diluted in PBS, membranes were incubated with secondary antibodies following the same staining protocol described in the ‘Immunostaining’ section. The horseradish peroxidase (HRP) substrate 3,3′,5,5′-Tetramethylbenzidine (TMB; Sigma-Aldrich, Merck Life Science UK Limited, Gillingham, UK) was added for 10 min. Images were acquired using the GelDoc RX+ system (BioRad Laboratories, Hemel Hempstead, UK).

### 4.7. Silver Staining

LPS was extracted using a bacterial LPS extraction kit (2B Scientific, Upper Heyford, UK) following manufacturer’s instructions. Briefly, cells were lysed using organic solutions, and the cell membrane phospholipids and proteins were disrupted and released in solution. LPS was then purified from the mixture with a high salt concentration solution and eluted in 10 mM Tris-HCl buffer (pH 8.0) (Sigma-Aldrich, Merck Life Science UK Limited, Gillingham, UK) after washing off salts with 70% ethanol. SDS-PAGE was performed as described in the ‘Western Blotting’ section, and gels were then silver stained using the SilverQuest Silver Staining Kit (Invitrogen, Paisley, UK) following manufacturer’s instructions. Images were acquired using the GelDoc RX+ system (BioRad Laboratories, Hemel Hempstead, UK).

### 4.8. Growth Studies

Bacteria were seeded into liquid LB medium cultures starting at OD = 0.1 and grown for 24 h. At regular intervals, samples were taken and analysed by spectrophotometry or by titration on agar plates. OD at A_600 nm_ was reported as a measure of growth density. Bacterial titers were measured by 10-fold serial dilutions prepared in sterile PBS; aliquots of three dilutions were plated in triplicate on LB agar plates supplemented with aromix and L-Tyr. The dilution range was selected to culture a reasonable number of bacteria to count per plate (between 30 and 300 colonies per plate). Plates were incubated at 37 °C for ≥18 h for colony counting, and colony-forming units (CFU) per ml were calculated by multiplying the average number of colonies on a plate by the corresponding dilution factor multiplied by 10.

### 4.9. Murine Immunogenicity

Bacteria for *in vivo* studies were grown at 37 °C in LB–aro up to the mid-exponential phase, harvested by centrifugation (4100× *g* at 4 °C for 20 min), washed in sterile cold PBS and concentrated to obtain a final cell density of approximately 6 × 10^10^ ± 0.5 × 10^10^ CFU/mL (based on OD_600 nm_ calculations) in PBS containing 10% glycerol (Sigma-Aldrich, Merck Life Science UK Limited, Gillingham, UK). These were frozen at −80 °C until needed. On the day of dosing, vials were thawed at room temperature and vortexed gently for several seconds to obtain a homogeneous suspension. Vials were centrifuged (4100× *g* at 4 °C for 20 min), supernatants removed, and pellets resuspended in 14 mL sterile cold PBS. This was repeated twice to ensure the pellet was washed three times in total, followed by a final resuspension in 1 mL sterile cold PBS.

Female Balb/c mice (purchased from Charles River, Margate, UK) at 6–8 weeks of age were maintained in sterilised ventilated cages with ad libitum access to food and water and with room conditions set at a temperature of 22 °C (±1 °C), 60% relative humidity and a 12 h light/dark cycle. Vaccines were diluted to the appropriate concentration in sterile cold PBS, followed by subcutaneous immunisation with 100 µL of solution equivalent to 1 × 10^8^ colony-forming units (CFU) of ZH9, 110^8^ CFU of ZH9PA and a 1:1 combination of 0.5 × 10^8^ CFU of ZH9 and 0.5 × 10^8^ CFU of ZH9PA. For combination injections, the vaccine strains were mixed, and a single injection was performed. Blood samples were collected on day 0 prior to immunisation via tail prick, and on day 35 or 42 following vaccination as a terminal bleed by cardiac puncture. Blood was left to coagulate on ice, and serum was separated by centrifugation for 5 min at 13,000 RPM. The supernatant was removed into a fresh Eppendorf tube, snap frozen and stored at −80 °C. All studies were performed at Evotec (Macclesfield, UK) under UK Home Office Licenses with local ethical committee clearance. All animal experiments were performed by experienced technicians that had completed the UK Home Office Personal License course and held current personal licenses.

### 4.10. ELISA

Antigens used in enzyme-linked immunosorbent assays (ELISAs) were LPS O:9 purified from *S.* Typhi ZH9 (The Native Antigen Company, Kidlington, UK) and LPS O:2 purified from attenuated *S.* Paratyphi A (generated by the Wellcome Sanger Institute, Hinxton, UK; purchased from The Native Antigen Company Kidlington, UK). Nunc F96 Maxisorp Immunoplates (ThermoFisher Scientific, Altrincham, UK) were coated with LPS antigens at an appropriate concentration for each batch after pilot experiments determined the optimal range of coating concentrations using control antibodies and checked batch purity. Mouse serum (collected as described above) was added to wells in serial dilutions. Pre-vaccination (d0) samples were pooled across mice to generate a negative assay control. Bound serum antibodies were detected with HRP-tagged secondary goat anti-mouse IgG antibodies (Sigma-Aldrich, Merck Life Science UK Limited, Gillingham, UK) and developed with TMB substrate (Sigma-Aldrich, Merck Life Science UK Limited, Gillingham, UK) for 10 min in the dark. The reaction was stopped with 1 M sulphuric acid, and absorbance was measured at 450 nm within 15 min of adding the Stop Solution with a Spark plate reader (Tecan Group Ltd., Männedorf, Switzerland). End-point titres were calculated by recording the dilution that intersected the curve at OD = 1 for each serum sample.

### 4.11. Statistical Analysis

Graphical data was plotted using GraphPad Prism 9 [42]. Data were statistically compared using either a one-way analysis of variance (ANOVA) with Tukey’s multiple comparisons test or a mixed-effects two-way ANOVA with a Bonferroni multiple comparisons test, where appropriate. Results with a *p*-value <0.05 were considered statistically significant.

## Figures and Tables

**Table 1:** Bacterial strains and plasmids used directly in this study.

Strain or Plasmid	Description	Source or Reference
**Strains**		
Top10 *E. coli*	F^–^ mcrA Δ(mrr-hsdRMS-mcrBC) φ80lacZΔM15 ΔlacX74 recA1 araD139 Δ(ara-leu)7697 galU galK λ^–^ rpsL(StrR) endA1 nupG	Life Technologies, Paisley, UK
*S*. Typhi ZH9	Ty2 Δ*aroC* Δ*ssaV*	Prokarium
*S*. Typhi ZH9PF	Ty2 ΔaroC ΔssaV Δ*t0918::SPA0911*	This work
*S*. Typhi ZH9PL2	Ty2 Δ*aroC* Δ*ssaV* Δ*rfbE*	This work
*S*. Typhi ZH9PA	Ty2 Δ*aroC* Δ*ssaV* Δ*rfbE::wbdR* Δ*t0918::SPA0911*	This work
**Plasmids**		
pBRT1Nc	Synthesised with Chloramphenical resistance gene	This work
pUCFlic2	Synthesised *S.* Paratyphi A *fliC*	This work
pUCWbdR	Synthesised *E. coli wbdR*	This work
pUCpF-difcat	Precursor *S.* Paratyphi A *fliC* integration Xer-cise plasmid	This work
pUCpW-difcat	Precursor *wbdR* integration Xer-cise plasmid	This work
pL2-difcat	Precursor *rfbE* deletion Xer-cise cassette	This work
pLGBK	Lambda Red helper plasmid	Prokarium

Abbreviations: *E. coli* = *Escherichia coli*; PCR = polymerase chain reaction.

**Table 2:** PCR and sequencing primers used in this study.

Primer Name	Primer Sequence (5′-3′)	Primer Function
5NotIdifcat	taagcggccgcATTTAACATAATATACATTATGCGCACCgcccgaacaccac	Primers designed to include NotI restriction sites at the 5′ and 3′ end respectively. The lowercase letters represent the region of homology to pBRT1Nc; the NotI restriction site is underlined and *dif* sites are in capital letters
3NotIdifcat	ggcggccgcGGTGCGCATAATGTATATTATGTTAAATgggcgagtttacatctcaaaaccg	
rfbE del F	AATAGGATGGAAAAGAGAGTTCTCTCTTGTTGATGCATTAACTGAAATAATTGAAGAGGAAGGGAAATGAAAAGCTTGGTACCGAGCTCG	*rfbE* deletion
rfbE del R	TTTGAAAGCCAAGAGGAAGCGGCAATAATAAGATGTCTTGGAATTCTAACCAACCTCAGTTTCCTCACTCTAGATGCATGCTCGAGCGGC	*rfbE* deletion
L1	AGGCTTGACTACAGAGCATTTAGATTATGTAG	Diagnostic primer for LPS locus
L6	ACATACTTCTACAATTAAGGAGTGAGAAGATTGATTATTAATACT	Diagnostic primer for LPS locus
L2	TCACGACTTACATCCTACTTCG	Diagnostic primer for LPS locus
L3	TGTTCCTGCCGGTATAACTG	Diagnostic primer for LPS locus
L4	CAGTTTCCTCACGTCAGCTT	Diagnostic primer for LPS locus
L5	CTGGCCATAATGCTTGTAATACCGCA	Diagnostic primer for LPS locus
F1	GCTGACTTGCGCATAAGCTTTGA	Diagnostic primer for Flagellin locus
F8	AACAGCCCTGCGTTAAATGAGT	Diagnostic primer for Flagellin locus
F3	TATTGCTCTGACGCTCAATG	Diagnostic primer for Flagellin locus
F6	ACGGTGATTTCTTTCATTACACAG	Diagnostic primer for Flagellin locus
F5	TTCAGCAGTATCAGCGCCGGT	Diagnostic primer for Flagellin locus

Abbreviations: *E. coli* = *Escherichia coli*; PCR = polymerase chain reaction.

## Data Availability

The original western blot, dot blot and silver staining data presented in this study are available as supplementary figures. All other data presented in this study are available on request from the corresponding author. The data are not publicly available due to the limited scope of the datasets.

## Supplementary Materials

The following are available online at https://www.mdpi.com/1422-0067/22/6/3287/s1.
