# Supplementary figures and images for: Engineering a Novel Bivalent Oral Vaccine against Enteric Fever
Source: Int J Mol Sci. 2021 Mar 23;22(6):3287. doi: 10.3390/ijms22063287 (PMC8005139; doi:10.3390/ijms22063287)

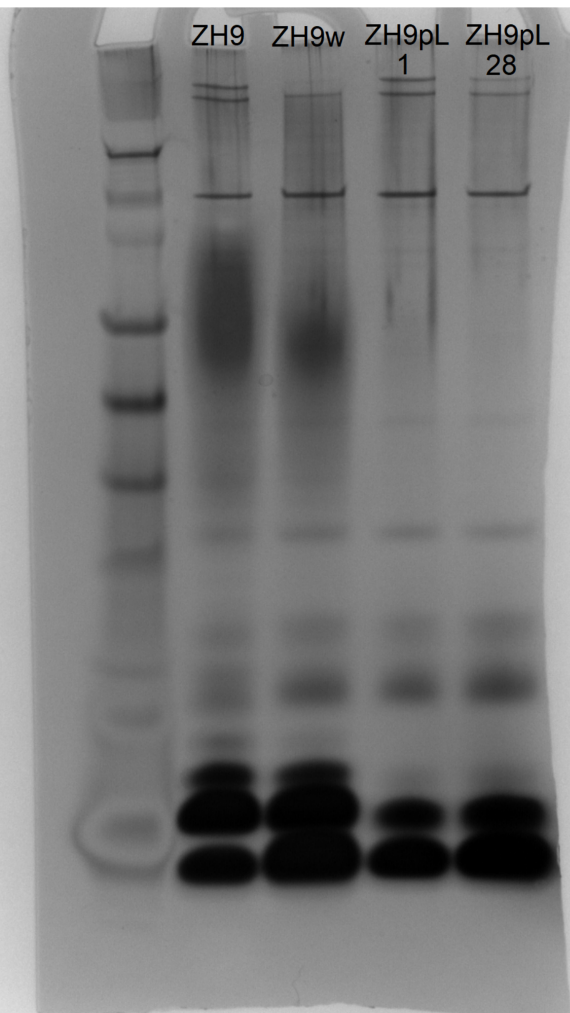

Supplement: Supplementary file 1 [file ijms-22-03287-s001.zip › Soulier_IJMS_Supplementary Figure 1.pdf]

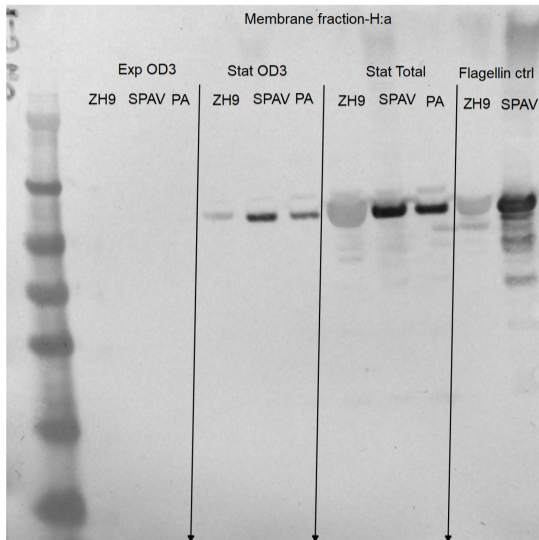

(a)

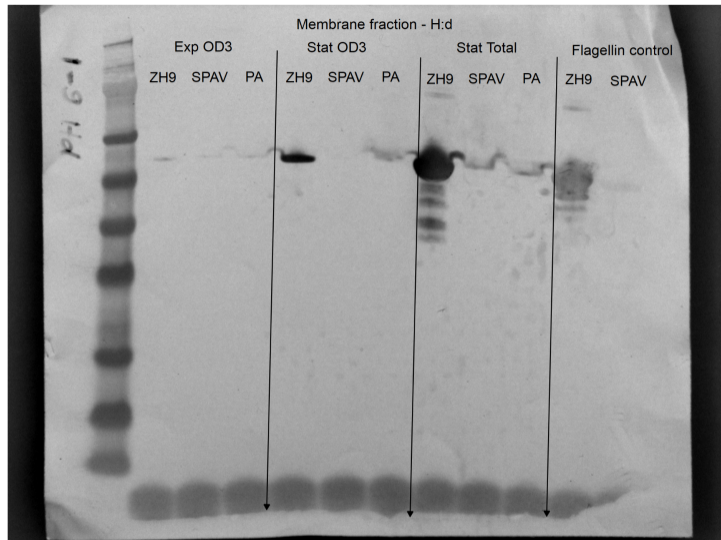

(b)

Supplement: Supplementary file 1 [file ijms-22-03287-s001.zip › Soulier_IJMS_Supplementary Figure 2.pdf]

Heat inactivated O/N culture

ZH9

ZH9PA

mAb S.Typhi LPS

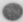

1

mAb S.Paratyphi A LPS

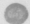

1

Supplement: Supplementary file 1 [file ijms-22-03287-s001.zip › Soulier_IJMS_Supplementary Figure 3.pdf]

Marker

ZH9

ZH9PA

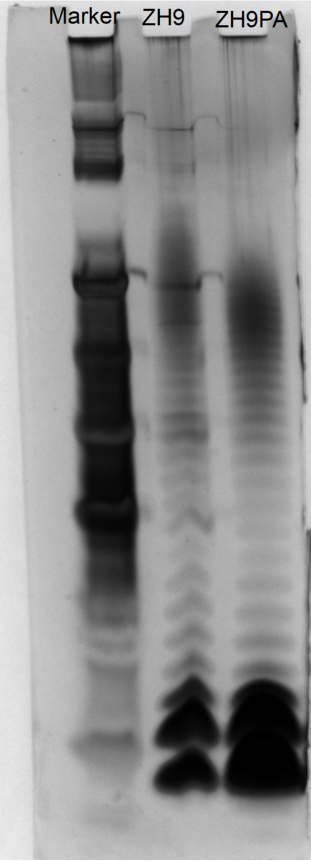

Supplement: Supplementary file 1 [file ijms-22-03287-s001.zip › Soulier_IJMS_Supplementary Figure 4.pdf]
